# Information on TikTok About the Oncogenic Potential of HPV in the Head and Neck: Implications for Public Health

**DOI:** 10.1007/s13187-025-02614-1

**Published:** 2025-04-01

**Authors:** Marina Maeso, Pia López-Jornet

**Affiliations:** Department of Dermatology, Stomatology, Radiology and Physical Medicine, Faculty of Medicine, University of Murcia, Hospital Morales Meseguer, Clinica Odontologica, Marques Velez S/N Murci, Murcia, 30008 Spain

**Keywords:** TikTok, Human papillomavirus, Oropharyngeal cancer

## Abstract

The aim of the present work was to conduct a descriptive cross-sectional study that consisted on the assessment of videos published in the social platform TikTok about HPV, and its appearance in the head and neck. The first 100 Spanish-language videos suggested by TikTok were selected, as well as the first 100 videos in English. To assess the reliability of the videos, the Modified DISCERN tool was utilized—Global Quality Score (GQS) for quality and modified DISCERN for reliability. Statistically significant differences were found between the variables language, with a significant relationship with objective (*p* = 0.01), current information (*p* < 0.01), and balance and objectivity (*p* = 0.01). The duration of the video was significantly related with the objective (*p* < 0.01) and clarity and understanding (*p* < 0.01), but not with other metrics such as the source of information (*p* = 0.87), or balance and objectivity (*p* = 0.92). The HPV contents must be verified by experts to avoid the propagation of incorrect information. There is little information available online about the relationship between oropharyngeal cancer and the human papillomavirus (HPV), which makes it difficult to access trustworthy and current resources about the subject.

## Introduction

The human papillomavirus (HPV) is associated with an increase in oropharyngeal cancer. Although most of the HPV infections are not diagnosed, as they are asymptomatic, it is estimated that 75% of the population of reproductive age has been exposed to HPV [1, 2]. There are around 200 genotypes of this virus, of which 26 have been associated with oral lesions. The most common low-risk genotypes, with respect to benign oral lesions, are HPV-6 and HPV-11, while HPV-16 and HPV-18 are associated with malignant lesions [1–8]. HPV is recognized as the most common sexually transmitted infection worldwide, affecting more than 11% of the global population. This infection can be subclinical, or may be manifested through benign lesions, such as such as squamous cell papilloma, condylomas acuminata, common warts, and focal epithelial hyperplasia [2–4].

HPV is not only the main cause of cervical cancer, but also an increasing number of head and neck cancers, such as oropharyngeal cancer (OPC). In the USA, the incidence of OPC has exceeded cervical cancer, becoming the most common cancer related with HPV [3, 4].

The types of HPV considered high risk and carcinogenic include genotypes 16, 18, 31, 33, 35, 39, 45, 51, 52, 56, 58, and 59. HPV-16 is responsible for 85 to 96% of the positive OPC cases due to HPV. The E6 and E7 viral proteins of HPV play a key role in the oncogenic power of the virus, as they block tumor suppression proteins such as p53 and the retinoblastoma protein (pRb), which leads to an uncontrolled cellular proliferation and a greater risk of cancer [5–7].

TikTok is a popular social network that has significantly grown in the past few years. Aside from being used as source of entertainment, it is also able to provide a wide range of content, providing health-related information, which spreads quickly and reaches a massive audience [8, 9].

Vaccines exist against HPV, which have significantly reduced the incidence of cancer. However, vaccination rates are still low. Despite the effectiveness of the vaccine against HPV, the reticence against vaccinations plays an important role in the dissemination of information about HPV [9–14]. Vaccine hesitancy on TikTok impacts the way information is shared, as distrust and doubts about vaccines influence which content is disseminated, what is commented upon, and how it is perceived. This allows misinformation to spread or causes the original message to become distorted [8].

The aim of the present work was to conduct a descriptive cross-sectional study that consisted on the assessment of videos published in the social platform TikTok about HPV, and its appearance in the head and neck.

## Methods

**Fig. 1:**
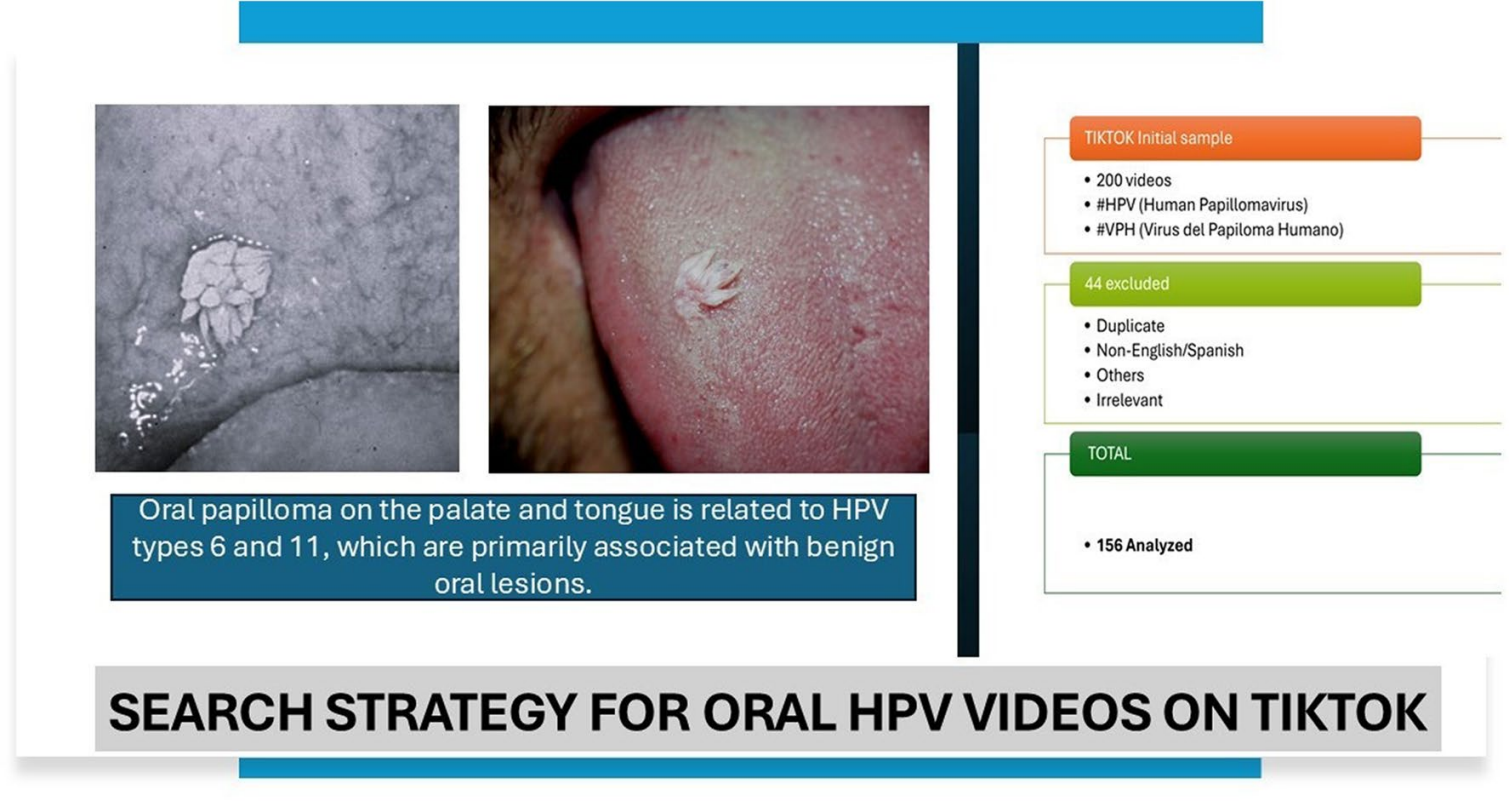
Flowchart of the study strategy

The first 100 Spanish-language videos suggested by TikTok were selected, as well as the first 100 videos in English and Spanish, by using the hashtags “HPV, oral and prevention, and cancer.” The videos that were not related with the HPV subject were excluded, as well as those that were duplicated, published in other languages other than Spanish or English, and videos without sound or with recording defects (Fig. 1).

To assess the reliability of the videos, the Modified DISCERN tool was utilized, granting 1 point per affirmative answer, and 0 for negative ones, following the method described in Kılınç DD et al. [15] Global Quality Score (GQS) for quality (Tables 1 and 2).

**Table 1:** Question Reliability Score (Adapted from DISCERN) (15)

Score	
1	Are the aims clear and achieved?
2	Are reliable sources of information used?
3	Is the information presented balanced and unbiased?
4	Are additional sources of information listed for patient reference?
5	Are areas of uncertainty mentioned?

Note: Modified DISCERN tool (1 point for answer “yes”, 0 points for answer “no”)

**Table 2:** GQS definition (Global Quality Score)

Score	
1	Poor quality, poor video flow, most information is missing, not useful for patients at all
2	Generally poor quality and flow, some information is provided but many important topics are missing, very limited usefulness for patients
3	Moderate quality, some important information is adequately discussed
4	Good quality and good flow, most relevant information is covered, useful for patients
5	Excellent quality and flow, very useful for patients

The following basic information was collected from each video: URL, duration, number of “likes,” comments, number of times shared, and number of saves. The videos were classified according to their main content into categories such as prevention, treatment, oral HPV, manifestations, diagnosis, transmission, oral cancer, and oropharyngeal cancer.

To assess the objectivity of the video scores, a second evaluator took part in the assessment, who independently scored 10% of the previously assessed videos. After the assessment, Cohen’s kappa coefficient was calculated, obtaining a value of 93.4%, which indicates a high degree of agreement between the evaluators.

In the statistical evaluation of the study, an initial descriptive analysis was performed to understand the distribution of the data and central trends. This was followed by the Shapiro–Wilk test to assess the normality of the data. For data that did not follow a normal distribution, the Kruskal–Wallis test was used to compare means across multiple groups, supplemented by post hoc Bonferroni adjustments to identify specific differences between groups. Relationships between variables were further explored using Spearman’s correlation for ordinal data and Fisher’s exact test for categorical data in cases of small sample sizes. A *p* value of less than 0.05 was considered significant. All statistical analyses were performed using R software version 4.3.1 (R Core Team, Vienna, Austria) [16].

## Results

Ultimately, 156 videos were assessed. With respect to the profiles of the content creators, 60% of the videos were created by doctors or dentists. In 39% of the videos, the profession of the creator was not identified, and 1% corresponded to testimonies not associated with the area of health. The subjects related with prevention and generalities were the most popular, as compared with subjects such as oropharyngeal/oral cancer, which obtained a significantly lower presence, with 7.6% of the total HPV subjects. In Tables 3 and 4, data related to the videos are presented, including their duration, as well as interaction metrics such as “Likes,” shares, and saves.

**Table 3:** TikTok content metrics: duration, likes, comments, and shares

Variable	Median	IQR	Minimum	Maximum
Duration (seconds)	51.0	59.0	5.0	351.0
Likes	157.0	1419.0	6.0	23,000.0
Comments	6.0	53.0	0.0	975.0
Number of times saved	22.0	182.0	0.0	6639.0
Number of times shared	15.0	101.0	0.0	1018.0

**Table 4:** Characteristics of the videos and information

Parameter	Objective (*p* value)	Source of information (*p* value)	Current information (*p* value)	Balance and objectivity (*p* value)	Clear and understandable (*p* value)	Support in decision making (*p* value)
Language	*p* = 0.01	*p* = 0.21	*p* < 0.01	*p* = 0.01	*p* = 0.06	*p* = 0.06
Duration	*p* < 0.01	*p* = 0.87	*p* = 0.81	*p* = 0.92	< 0.01	< 0.01
Likes	*p* = 0.05	*p* = 0.06	*p* = *0.62*	*p* = 0.42	*p* = 0.23	*p* = 0.16
Comments	*p* = 0.06	*p* = 0.01	*p* = 0.73	*p* = 0.36	*p* = 0.02	*p* = 0.25
Number of times saved	*p* = 0.12	*p* = 0.13	*p* = 0.02	*p* = 0.02	*p* = 0.05	*p* = 0.19
Number of times shared	*p* = 0.12	*p* = 0.14	*p* = 0.11	*p* = 0.16	*p* = − 0.12	*p* = 0.26

**Fig. 2:**
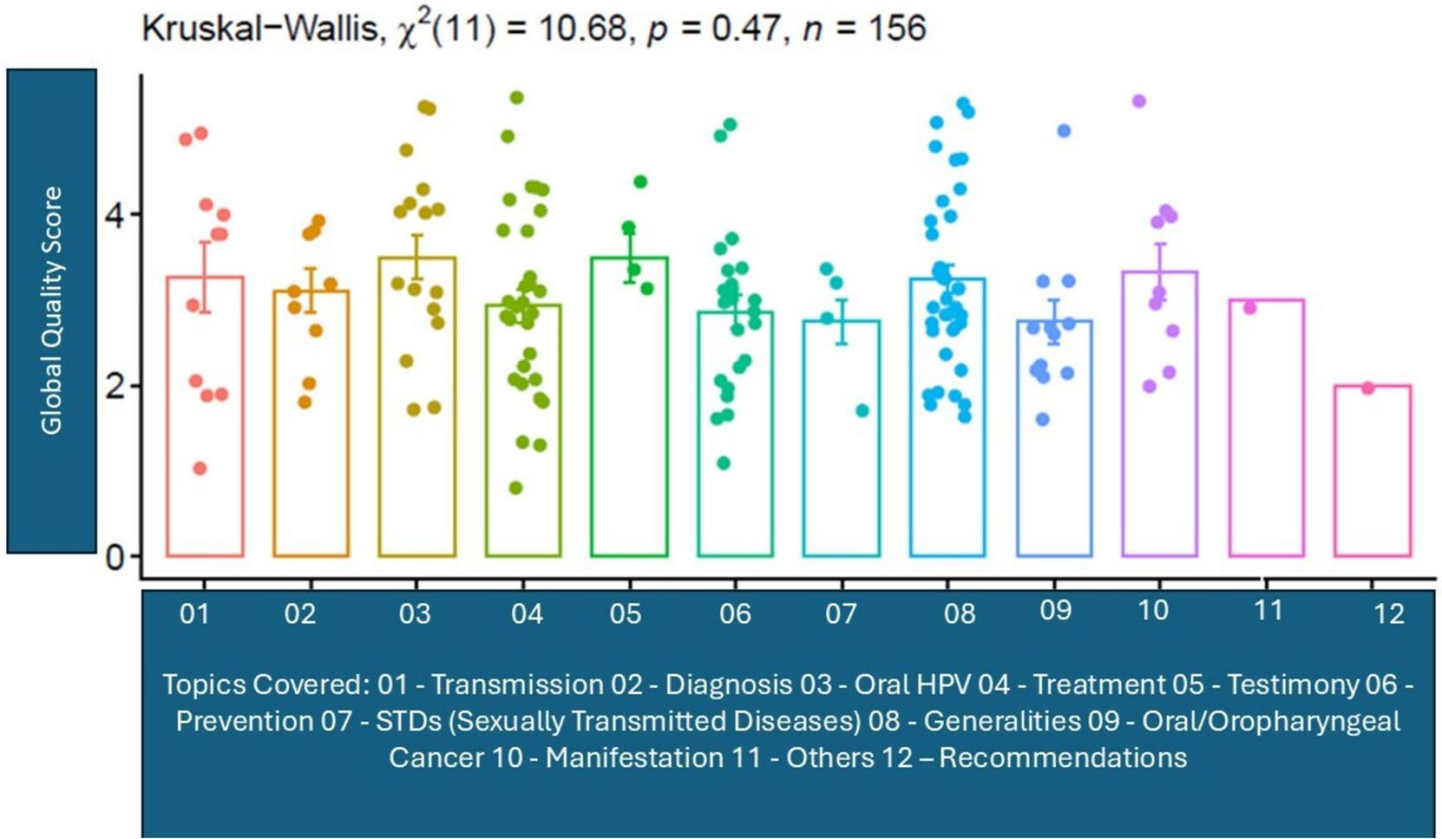
The overall quality related to the topics addressed about HPV

Assessment of content reliability: the average reliability score of the videos, as evaluated by the Modified DISCERN, was 3.49 ± 0.35 (where 1 is the minimum score and 5 is the maximum one). It is important to highlight that most of the videos did not reveal conflicts of interest or funding sources (96.79%). The overall quality related to the topics addressed about HPV is reflected in Fig. 2.

Statistically significant differences were found between the variables language, with a significant relationship with objective (*p* = 0.01), current information (*p* < 0.01), and balance and objectivity (*p* = 0.01). The duration of the video was significantly related with the objective (*p* < 0.01) and clarity and understanding (*p* < 0.01), but not with other metrics such as the source of information (*p* = 0.87), or balance and objectivity (*p* = 0.92).

No significant correlations were found between the duration of the video and interaction metrics such as likes, comments, how many times it was saved, or shared (*p* > 0.05) (Table 1).

## Discussion

The proportion of oropharyngeal cancer attributed to HPV has increased, even exceeding tobacco and the consumption of alcohol as the most common risk factor [2, 4]. Some studies have demonstrated that an increasing number of patients resort to social networks to seek information about health matters [9, 10]. This underlines the need to complement the information provided by clinics with clear and precise online resources. Disinformation about health, however, is disseminated faster than evidence-based information, which is an important challenge. TikTok has the power to influence the content consumed by its users, which makes it a powerful platform for health education. It is vital for HPV content to be created or validated by health professionals to guarantee that the information is precise [9, 10].

One of the most worrying findings is that 92.95% of the publications did not share bibliographical references or studies that supported the credibility of the information. This underlines the need for the TikTok contents to be supervised by experts on the subject. In addition, it would be valuable to integrate health professional societies into the platform, as well as institutions and health organizations, to guarantee quality content that offers education that is complete and adequate [7], to thereby guarantee optimal content that ensures an ideal and complete health education.

The findings of Boatman et al. [8] and Massey et al. [10] highlight the key role of social media in shaping public perception of the HPV vaccine. Boatman et al. identify TikTok as an emerging platform for disseminating vaccine-related messages, noting that antivaccine videos, which focus on side effects, generate more engagement than provaccine content, which emphasizes cancer prevention. On the other hand, Massey demonstrates that misinformation on Instagram spreads mainly through personal narratives and unfounded claims about risks. Although both studies analyze different platforms, they converge on the need for targeted strategies to counter misinformation and promote evidence-based information.

In our analysis, the existence of significant difference was observed between the duration of the videos and the clarity of the messages perceived (*p* < 0.01). The longer videos allowed the creator to better develop their content, which made it possible to convey a clearer message to the audience. This suggests that in order to improve the quality of information in TikTok, the content creators must consider lengthening the duration of the videos to offer more complete explanations.

Another of the weaknesses of TikTok is its dynamic nature, with a fast rotation of content, which can limit the permanence and visibility of the educational messages [9]. In addition, the analysis of the video contents does not allow for a deep reflection of how the spectators process and use this information [10]. Therefore, future studies should focus on how TikTok users understand and apply the HPV information they consume.

A greater transparency in the verification of credentials of the content creators in platforms such as TikTok could improve credibility and help users to make more informed decisions about the health information they consume. On the other hand, healthcare providers must play an active role in HPV education, by promoting safe sexual practices, quitting smoking, and by promoting early vaccination to reduce the risk of illnesses related with HPV [7, 12].

This study has some inherent limitations, which have also been acknowledged in previous research, including that of Kılınç DD [15]. First, as a cross-sectional study, the analysis is confined to a single point in time, potentially affecting the generalizability of the results. Second, the evaluation frameworks utilized, Global Quality Score GQS and DISCERN, are based on subjective assessments, which may introduce variability in interpretation. Third, the ever-evolving nature of digital content, with an overwhelming volume of uploads and views, poses challenges in capturing a truly representative database.

In conclusion, the HPV contents must be verified by experts to avoid the propagation of incorrect information. There is little information available online about the relationship between oropharyngeal cancer and the human papillomavirus (HPV), which makes it difficult to access trustworthy and current resources about the subject. Further research is needed on how users process and react to information on TikTok.

## Data Availability

The data will be available upon request and with the author’s authorization.
